# Dyeing to live – a case of clozapine in disguise, and physicians' courage

**DOI:** 10.1192/bjo.2021.347

**Published:** 2021-06-18

**Authors:** Alma Rae, Thomas Kinghorn

**Affiliations:** 1 Locum Consultant Psychiatrist; 2 NHS Lothian

## Abstract

**Objective:**

… in which clozapine tablets were dyed pink, to work around a delusion preventing treatment, and physicians tolerated and monitored an alarming early response to the drug.

**Patient:**

56-year-old female with severe enduring Bipolar I Disorder, current episode manic with psychosis, already an inpatient for six months. When first seen by us, polypharmacy was evident including haloperidol 25 mg daily. Thorough trials of mood stablisers and second generation antipsychotics in various combinations had all failed. She had never had a clozapine trial.

**MSE:**

Dishevelled middle-aged woman of European descent. Restless; shuffling gait; speech pressured, rapid and whispering, often to the point of unintelligibility. Affect labile: anxious and distressed, suspicious, angry, elevated and demanding. Thought form tangential+++ content paranoid persecutory themes, preoccupied with sexual trauma and delusional belief that yellow medication whether solid or liquid was poisonous. Risks of vulnerability, falls, aggression, neuroleptic malignant syndrome (NMS) and protracted psychotic mania requiring long term hospitalisation.

**Plan:**

Change to clozapine.

**Problem:**

All formulations are yellow.

**Solution:**

Team discussion, ethical analysis, clozapine tablets dyed with red vegetable dye.

**Ethical analysis:**

Potential benefit to patient great; current medications not effective and NMS possibly developing; she was fully informed about clozapine with no attempt made to hide the identity of the now crimson tablets.

**Outcome:**

Patient accepted the clozapine. Temperature, C reactive protein (CRP) and troponin were all normal at baseline but all rose above normal in week 1 of initiation. They peaked in week 3 and by week 4 were dropping, normalising completely within a few weeks. She was transferred to a medical ward for monitoring during weeks 2 and 3 of titration. There were no electrocardiogram changes, no chest pain, no signs of bowel obstruction and no evidence of agranulocytosis. Clinically, she remained well throughout except for the rise in temperature. Once the yellow medicine delusion receded she accepted undyed yellow tablets; the result was discharge home with her best mental state and level of functioning in 15 years.

**Significance of this case:**

There are no cases in the literature that we could find where tablets had been dyed, or where clozapine had been persisted with when such rises in temperature, CRP and troponin occurred. This case illustrates both. The risks in our view were outweighed by the simple fact that clozapine was her only hope of a life worth living.

